# The use of health care during the SARS-CoV-2 pandemic: repeated cross-sectional survey of the adult Swiss general population

**DOI:** 10.1186/s12889-021-10854-1

**Published:** 2021-05-03

**Authors:** Stéphanie Giezendanner, Roland Fischer, Laura Diaz Hernandez, Andreas Zeller

**Affiliations:** Centre for Primary Health Care, University of Basel, Kantonsspital Baselland, Rheinstrasse 26, 4410 Liestal, Switzerland

**Keywords:** SARS-CoV-2, Pandemic, COVID-19, Swiss, Health care, Primary care, General practitioner, Ecology of medical care

## Abstract

**Background:**

The distribution of health care resources during a pandemic is challenging. The aim of the study was to describe the use of health care in a representative sample of the Swiss population during the SARS-CoV-2 pandemic in 2020, and to compare it to data from a survey conducted in 2018.

**Methods:**

We conducted an observational, population-based, nationwide, repeated cross-sectional survey of the adult Swiss general population in 2018 and in March and April 2020 during the first wave of the SARS-CoV-2 pandemic. Recruitment and data acquisition was conducted by the Link Institute in Lucerne in representative samples of Swiss citizens in 2020 and in 2018. Variables of interest were estimates of health problems, health seeking behaviour, medication and health care use in the population.

**Results:**

In total, we included data of 1980 individuals (in 2018 *N* = 958 and in 2020 *N* = 1022). Across both rounds of data collection the median age was 46 years (range = 18–79 years) and 50% were women. Per 1000 adults, half had at least one symptom and a quarter sought medical advice across both surveys. The most frequently consulted health providers in 2020 were general practitioners (GP) (180/1000), specialist physicians (41/1000), pharmacies (38/1000), the internet (26/1000) and accident and emergency units (25/1000). Compared to 2018, we noted a significant increase in the use of health providers during the pandemic, which was independent of demographic variables for the following health care providers: use of internet (OR = 9.8), pharmacy (OR = 2.64), accident and emergency units (OR = 2.54), and a significant decrease in the number of people who consulted specialist physicians (OR = 0.46). Overall, 76/1000 contacted their GP in relation to COVID-19.

**Conclusions:**

Compared to 2018, GPs remained the most important source of medical advice for the population during the first wave of the COVID-19 pandemic in Switzerland. While the self-appraisal of health problems and of the need for medical advice remained constant, individuals seemed to change their provider choice during the pandemic, with an increased utilisation of accident and emergency units and pharmacies, which represent easily accessible and low-threshold medical services.

**Supplementary Information:**

The online version contains supplementary material available at 10.1186/s12889-021-10854-1.

## Background

Severe acute respiratory syndrome coronavirus 2 (SARS-CoV-2) is a novel coronavirus first detected in Wuhan, in the Chinese Province of Hubei [1]. The World Health Organisation labelled the disease caused by SARS-CoV-2 coronavirus disease 2019 (COVID-19) and officially declared COVID-19 on March 11th 2020 a pandemic [2]. Since the initial detection of the virus, over 88 million cases of COVID-19 and over 1.9 million deaths have been confirmed worldwide (as on January 12th 2021) [3]. The first case in Switzerland was reported on February, 24th in the Canton of Ticino. As of June 16th, 2020, the incidence of the disease was 363 cases per 100′000 habitants [4] (see Supplementary file 1 for cumulative sum of COVID-19 cases in Switzerland). However, the incidence was much higher in the French and Italian language regions where incidences ranged from 286 per 100,000 (Canton of Jura) to 1051 per 100,000 (Canton of Geneva). In the Italian speaking part of Switzerland (Canton of Ticino), an incidence of 931 per 100,000 was reported. In the German language region, the incidence ranged from 100 per 100,000 (Canton of Schaffhausen) to 583 per 100,000 (Canton of Basel City).

Due to the rapid spread of SARS-CoV-2 in Switzerland, the Federal Office of Public Health enacted lockdown laws relating to movement, gatherings, high street business operations, and closing schools on March 16th 2020. In addition, a national campaign to provide COVID-19 hygiene guidance and several telephone hotlines were launched. Restrictions in the health care system were that cantons could compel private hospitals and clinics to increase their bed capacities for COVID-19 patients and that health care facilities such as hospitals and clinics, medical practices and dental practices were not allowed to offer non urgent medical interventions and therapies. As a consequence of these restrictions, while primary care physicians and paediatricians were allowed to provide part of their usual patient care, most specialists were forced to close their practices, and hospitals stopped all non-urgent patient care, such as elective surgery. One key message of the Federal Office of Public Health was that patients should call their general practitioners (GPs) to discuss further necessary action with regards to symptoms, risk factors, going to work, and referral for testing or hospitalisation [5]. In urban areas, people were recommended to get tested for COVID-19 at walk-through testing centres. In remote areas, testing was performed at GPs’ practices. Mobile test teams were established specifically for nursing homes. The cantonal health authorities repeatedly updated comprehensive clinical guidance for GPs and the population was regularly informed by the media (radio, television, newspapers) and announcements in public places.

It is important to understand how health care is organized in Switzerland. Health insurance is mandatory in Switzerland and encompasses the standard model with the fee for service plans (currently 28% of insured), and since the early 1990s also alternative gatekeeping plans (currently 72% of insured) [6]. The Swiss healthcare system is a combination of public, subsidised private and totally private systems. Primary medical care is delivered mainly by GPs practicing in private practice [7]. There are 40% primary care physicians and 60% specialists in ambulatory care in Switzerland [7]. During a non-pandemic situation, the primary care sector has an important role in treating and controlling infectious diseases [8]. If a pandemic emerges, GPs will treat patients during its complete course. In addition to insufficient knowledge, a rapidly changing flood of information, and in part contradicting statements in the public media and world wide web [9], a pandemic situation by itself inevitably triggers fears, worries, and uncertainty in the population. At this point, the GPs’ role to provide sound recommendations, reduce psychological distress, and break infection chains of the virus is crucial. Seeking primary care services can increase in such a scenario, driven in part by reluctance to seek care in a hospital setting and in part by health authorities’ designation of GPs as one of the first points of contact when people develop symptoms [10]. Remote consultations with the GPs are becoming daily practice, requiring doctors to adapt the way they interact with patients (via telephone or video) to assess them [11]. As such, the role and scope of primary care expands under these circumstances. GPs are also essential to prevent patients from overfilling emergency rooms and ultimately to reduce hospitalisation rates. GPs are in a pivotal position to direct the population’s use of the health care system and to support a reasonable and fair allocation of health care resources.

To investigate health care utilisation in Switzerland (outside a pandemic), our research group conducted a nationwide evaluation in 2018 using the population ecology theory [12]. This concept is a valuable tool for researchers and health care policy makers to estimate the healthcare behaviour of a population in order to guarantee the fair distribution of health care resources. It aims to measure and reflect the quantity, quality and distribution of health care services. The ecology of care model was first introduced by White et al. [13] and was replicated in different countries around the globe [14–20]. The key findings of our recent study showed that outside a pandemic situation, GPs were the most important source of medical advice for the population in Switzerland [12].

Health care resource allocation is already a challenging task that becomes even more critical during an epidemic situation in order to provide appropriate medical care and keep morbidity and mortality rates as low as possible. A majority of patients infected by SARS-CoV-2 show mild forms of the disease [21] that can be handled outside hospital settings [22]. The management of these patients is a fundamental task for GPs, which relieves hospitals and allows intensive care medical services to be allocated to severely ill patients who truly need tertiary care.

The 2020 COVID-19 pandemic offers a unique opportunity to investigate the health care behaviour and the potentially higher prevalence of illness in the population. It also offers the exceptional opportunity to compare the use of the health care system to “normal” conditions. The aim of the study was to evaluate the health seeking behaviour of a representative sample of the Swiss population in a two-month period during the SARS-CoV-2 pandemic in March and April 2020, and to compare it with the use of health care of Swiss individuals outside a pandemic in 2018.

## Methods

### Data collection

A representative sample of the Swiss population was surveyed in 2018 and in 2020 [23].

The LINK Institute Lucerne, Switzerland (https://www.link.ch/) selected adults from their panel and conducted the interviews on our behalf. In the 2018 survey, people aged 18 years or older were interviewed by telephone (for details, see [12]). Because of disease control measures during the pandemic in the 2020 survey, it was not possible to perform a telephone survey. A web-based survey was performed instead, and included people between 18 and 79 years.

In the 2020 survey, the contacts were randomly drawn from the LINK online panel for each quote (e.g. quote 1 = German-speaking Switzerland, male, between 18 and 29). This panel consists of 115,000 active members who are representative of the Swiss population in terms of age, sex and language region. Due to the implementation in this online panel, all contacts were eligible. All participants regularly take part in studies and incorrect or ineligible contacts are continuously removed. No self-selection is possible in the panel, this means that participants cannot register for studies with the LINK Institute, but are specifically contacted and asked by the LINK institute to take part. For each quota, an additional 5% was added as a buffer in order to see which quotas are already filled. LINK did not exclude contacts, but worked with Rim weighting (see below). Quotas that were on overflow were weighted down and adjusted accordingly.

### Informed consent for survey participation and ethical approval

The need for ethics approval was waived by the Ethics Committee of Northwest and Central Switzerland (EKNZ) (Project-ID: Req-2020-00449), since the survey complies with the general ethical principles for human research. Verbal informed consent to participate in the survey was obtained before the telephone interview and implicit consent was assumed when a participant completed and returned the web-based survey. Information was collected anonymously from all participants who agreed to take part in this study.

### Questionnaire

The questionnaire was fully structured with semi-open and closed questions (see Supplementary file 2).

Respondents indicated the presence of any health problems in the last 2 months, whether and where they asked for medical advice (specifically: advice from the internet, family or friends, a drug store, a pharmacy, a telephone medical advice centre, a general practitioner, specialist physicians, an accident and emergency unit, an outpatient or poli-clinic, a physiotherapist, a dentist or a practitioner of alternative medicine. New questions in 2020 comprised the use of psychologist and psychiatrist services and federal or cantonal COVID-19 hotlines. Respondents also indicated whether they were hospitalised within the last 2 months as well as details regarding their in-hospital and post-hospital care.

### Sample size

With 1000 interviews, the margin of error lies within +/− 3% at a 95% confidence interval. The power to detect a difference in rare events decreases rapidly. This is why we only considered events occurring at > 20/1000.

### Demographic weighting

To match the survey sample with the specified target of the Swiss population [23, 24], weighting was performed by the LINK Institute Lucerne after sample collection using raking also known as random iterative method (rim) weighting [25] on demographic characteristics (language region, sex, age category, employment status and household-size).

### Analyses

All analyses are conducted on the weighted samples using the R package “survey” [26], which weights each observation by the inverse of its sampling probability. In particular, we used functions such as “svytable” to compute weighted cross-tabulations, “svyciprop” to calculate the confidence intervals for proportions using the “likelihood” method and “svychisq” to test for significant associations between two categorical variables. Further, we compared different measures of health care use (binary outcome) between pandemic and non-pandemic times (predictor) by fitting a generalised linear model of the binomial family to the data with inverse-probability weighting and design-based standard errors using the function “svyglm”. Moreover, in multivariable logistic regression models we tested the association of survey year (2020 vs. 2018) on measures of health care use (binary outcome) controlled for demographic variables (continuous predictors: age and household size, categorical predictor: sex, employment status, language region).

### Subgroup analyses

Subgroup analyses for variables of interests in health and health care use were conducted for the 2020 survey comparing men and women, four age groups, three language regions, rural and urban areas, household size (one, two or three or more household members), as well as employed and unemployed people.

## Results

### Participants

In the 2018 survey, a total of 1025 people participated and were described in detail in a previous publication [12]. In the 2020 survey, from 4110 initially contacted people, 1050 (25.5%) participated and 1022 (24.9%) completed the web-based survey. Because the 2020 survey did not include any people in the age category > 80 years, 63 participants from this age category from the 2018 survey were excluded from the current analysis. In total, 958 and 1022 participants from the 2018 and 2020 surveys were included in the current analysis respectively.

### Demographic characteristics

In 2018 and 2020, the median age of the weighted sample was 49 (IQR = 38–59) years and 45 (IQR 33 to 58) years respectively. Approximately half of respondents were women, the majority were German-speaking (72%), a quarter were French-speaking (24%) and 4% were Italian-speaking.

Table 1 shows the weighted demographic characteristics across both surveys. Age groups, sex and language regions did not differ between the two surveys. However, we observed significant differences in household size, area type and employment status across surveys. The vast majority (95% in 2018, 90% in 2020) of participants were registered with a general practitioner.

**Table 1:** Weighted total sample numbers for sex, age group, language region, employment status, household size and urban/rural area in Switzerland by survey year 2018 and 2020

Variables	2018 (***N*** = 958)			2020 (***N*** = 1022)					
	Estimate per 1000	Lower CI	Upper CI	Estimate per 1000	Lower CI	Upper CI	χ^**2**^	df	***P***-value
Sex									
Women	500	448	553	490	453	528	0.15	1.00	0.701
Men	499	444	554	510	472	547			
Age group									
18–29 years	192	155	228	208	182	234	1.32	2.97	0.266
30–44 years	273	227	318	291	261	321			
45–59 years	292	250	333	299	268	331			
60–79 years	244	211	277	201	175	228			
Language region									
German	714	651	778	720	678	762	0.06	1.98	0.940
Italian	43	29	57	40	28	52			
French	242	204	280	240	212	268			
Household size									
1 person	325	265	386	186	161	212	20.98	1.91	< 0.001
2 people	334	292	375	356	323	389			
3 and more people	341	313	369	456	419	492			
Area type									
Rural	276	238	314	218	191	245	7.06	1.00	0.008
Urban	724	659	788	782	739	824			
Employment									
Employed	707	641	774	745	704	787	3.59	1.00	0.058
Not employed	292	258	326	250	221	279			
Registered with a GP	947	878	1016	901	858	944	11.79	1	< 0.001

*CI* 95% confidence interval, *df* degrees of freedom. There was no missing data except for household size (0.20% in 2020) and employment status (0.49% in 2020)

### Medical ecology in terms of type of care

**Fig. 1:**
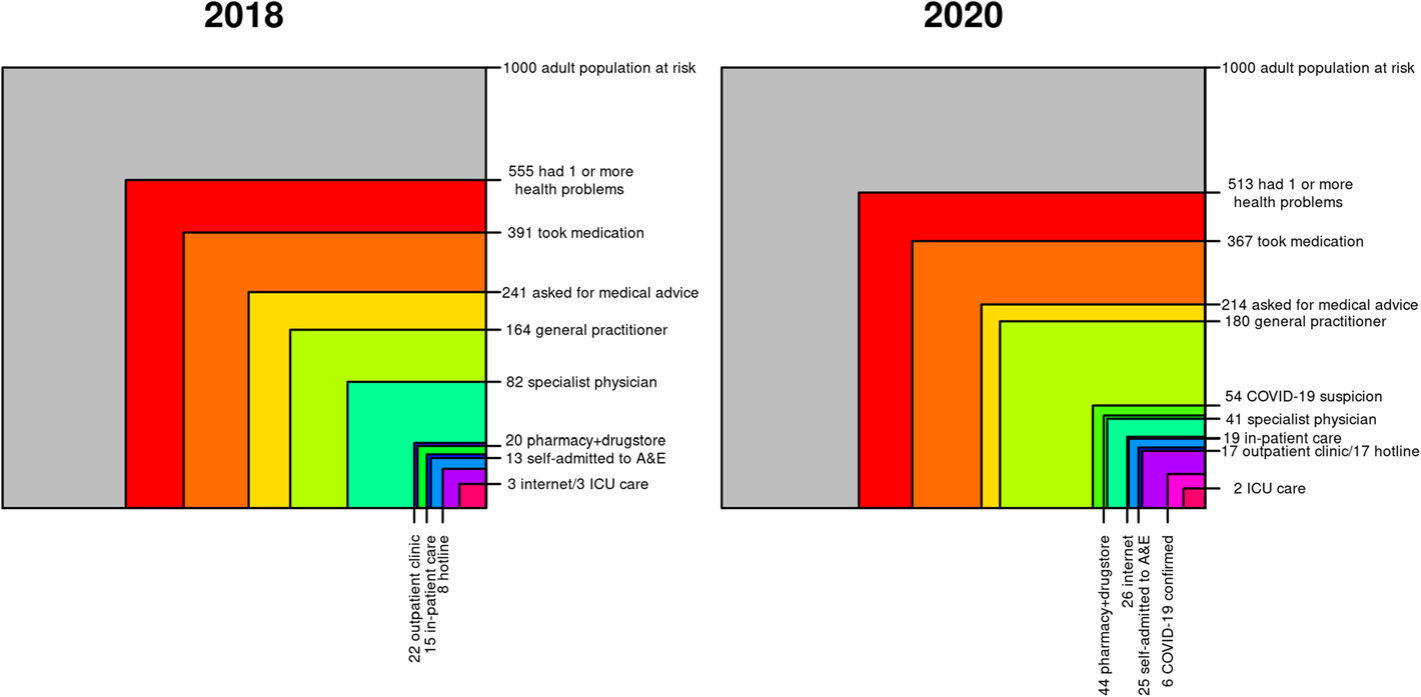
Sources of health advice. * *p*-value < 0.05

The unadjusted, bimonthly, weighted rates of health problems and health care use among Swiss residents across both survey years are reported in Table 2 and Fig. 1. We found that the frequency of health problems, seeking medical advice and medication did not differ significantly between surveys. Per 1000 adults, half had at least one symptom, a quarter sought medical advice and approximately a third took medication. The health care providers most often contacted in 2020 were GPs (180/1000), specialist physicians (41/1000), pharmacies (38/1000), accident and emergency departments (25/1000), and outpatient clinics (17/1000). Some people consulted the internet (26/1000) or national or cantonal Corona hotlines (6/1000). During the pandemic, 76 (95% CI =61, 93) out of 1000 contacted their GP in relation to COVID-19. Of those, most people used the phone (77%), while the minority went to the practice in person (14%), wrote a mail (7%) or a short text message (3%).

**Table 2:** Medical ecology in terms of type of care (sources of health advice, hospital or post-hospital care) by survey year 2018 and 2020

	Survey 2020 (*N* = 1022)			Survey 2018 (*N* = 958)				
	Estimate per 1000	Lower CI	Upper CI	Estimate per 1000	Lower CI	Upper CI	Statistic^a^	*p*-value^a^
**Health problems**								
had 1 or more health problems	513	482	543	555	514	595	−1.62	0.106
one	326	304	347	371	340	400	−0.92	0.356
more than one	184	163	205	182	153	213	0.92	0.356
took medication	367	347	386	391	358	421	0.47	0.637
asked for medical advice	214	192	235	241	210	274	−0.48	0.629
**Sources of health advice**								
general practitioner ^b^	180	159	201	164	136	195	1.26	0.208
specialist physician	41	31	53	82	62	103	−3.02	0.003
pharmacy	38	28	50	18	7	36	2.12	0.035
internet	26	18	36	3	1	9	3.74	< 0.001
accident and emergency unit	25	17	36	13	6	24	2.13	0.034
outpatient clinic	17	10	26	22	12	35	−0.32	0.750
psychotherapist or psychologist	15	9	23					
family/friends	14	8	22	6	2	14	1.68	0.094
physiotherapy	12	6	19	3	1	7	2.59	0.010
CAM practitioner	12	6	19	7	2	15	1.24	0.216
telephone medical advice center	11	6	18	8	2	19	0.73	0.464
drugstore	6	2	12	2	0	7	1.37	0.171
federal/cantonal COVID-19 hotline	6	2	12					
dentist	5	2	11	6	2	16	−0.18	0.859
other	4	1	9	5	1	14	−0.05	0.957
no answer	1	0	5	0	0	2	0.65	0.514
**Hospital or post-hospital care**								
had in-patient hospital care	19	12	29	15	9	24	0.89	0.373
Levels of care								
in normal care unit	17	13	19	13	10	14	0.69	0.497
in intensive care unit	2	0	6	3	1	5	−0.69	0.497
had a surgical procedure	8	4	12	6	4	9	0.05	0.958
needed mechanic ventilation	0	0	0					
Levels of hospital								
were in university hospital	8	4	12	5	3	8	0.41	0.686
were in cantonal hospital	5	2	10	2	1	4	1.21	0.233
were in private hospital	4	1	8	2	0	4	0.97	0.338
were in regional hospital	2	0	6	7	4	10	−2.11	0.041
Post-hospital care								
were in rehabilitation clinic	5	2	9	4	2	7	0.09	0.925
had ambulatory nursing care	2	0	6	3	1	6	−0.89	0.379

CAM = complementary and alternative medicine

^a^from univariable logistic regression with survey year (2020 vs 2018) as predictor

^b^ Including contacts with the GP due to COVID-19 questions

Confounder-adjusted estimates of health care use during a pandemic can be found in Table 3. Corrected for demographic variables, we noted a significant increase in the use of health providers during the pandemic; in particular in the use of the internet (OR = 9.8, 95%CI = 2.93–31.31), physiotherapy services (OR = 4.43, 95%CI = 1.51–19.91), a pharmacy (OR = 2.64, 95% CI = 1.08–6.59), accident and emergency departments (OR = 2.54, 95% CI = 1.07–4.98), and a significant decrease of people who consulted specialist physicians (OR = 0.46, 95%CI = 0.28–0.77) compared to 2018. The adjusted multivariable models showing the effect of all demographic variables can be found in the Supplementary Material (see Supplementary file 3).

**Table 3:** Multivariable logistic regression model of the association of a pandemic situation with health problems, medical advice seeking and hospitalisation

	unadjusted analyses			adjusted analyses			
	OR	Lower 95% CI	Upper 95% CI	Adj. OR	Lower 95% CI	Upper 95% CI	***p***-value
**Health problems**							
one or more health problems	0.84	0.69	1.04	0.85	0.69	1.04	0.106
asked for medical advice	0.93	0.7	1.24	1.01	0.76	1.34	0.950
**Sources of health advice**							
general practitioner	1.22	0.90	1.66	1.31	0.96	1.77	0.087
specialist physician	0.47	0.28	0.77	0.46	0.28	0.75	0.002
pharmacy	2.66	1.08	6.59	2.64	1.08	6.45	0.034
internet	9.57	2.93	31.31	9.80	2.90	33.09	0.000
accident and emergency unit	2.31	1.07	4.98	0.46	0.28	0.75	0.002
outpatient clinic	0.89	0.42	1.85	0.91	0.41	2.00	0.805
family/friends	2.45	0.86	6.98	2.15	0.72	6.39	0.171
physiotherapy	5.49	1.51	19.91	4.43	1.11	17.72	0.036
CAM practitioner	2.01	0.67	6.06	1.53	0.48	4.93	0.475
telephone medical advice center	1.55	0.48	5.00	1.87	0.63	5.54	0.259
drugstore	3.41	0.59	19.65	2.82	0.40	19.74	0.297
dentist	0.88	0.22	3.56	0.96	0.26	3.57	0.947
other	0.96	0.18	5.04	1.11	0.19	6.46	0.911
no answer	2.53	0.16	40.76	4.69	0.06	370.59	0.489
**Hospital or post-hospital care**							
had in-patient hospital care	1.36	0.69	2.66	1.45	0.71	2.95	0.311
Levels of care							
in normal care unit	0.52	0.08	3.33	0.65	0.09	4.97	0.682
in intensive care unit	1.91	0.30	12.20	1.53	0.20	11.71	0.682
had a surgical procedure	1.04	0.28	3.83	0.37	0.03	4.11	0.427
Post-hospital care							
were in rehabilitation clinic	1.07	0.24	4.80	0.81	0.11	5.98	0.840
had ambulatory nursing care	0.42	0.06	2.82	0.00^b^	NA^b^	NA^b^	NA^b^

*CAM* Complementary and alternative medicine, *OR* Odds ratio, *CI* 95% confidence interval. Adjusted OR were controlled for household size, urban/rural region, employment status, sex, age and language region. *P*-values of the univariable regression are reported in Table 2

^a^ Including contacts with the GP due to COVID-19 questions

^b^GLM for levels of hospitals and ambulatory nursing care not possible because fitted probabilities numerically 0 occurred

Subgroup analyses showing comparisons of health care use across demographic variables are presented within the Supplementary Material (see Supplementary files 4, 5, 6, 7, 8 and 9).

## Discussion

During the first wave of the COVID-19 pandemic Swiss residents reported a similar frequency of health problems, compared to a sample drawn in 2018. The GPs remained the primary source of health advice. Respondents reported lower utilisation of health advice from specialist physicians, but reported higher utilisation of pharmacies, physiotherapy and the internet during the pandemic. Accident and emergency units were also consulted more frequently, but in-patient hospital care did not differ significantly before and during the pandemic. Only 6 out of 1000 residents contacted federal or cantonal COVID-19 telephone hotlines.

GPs are the most frequent source of health advice outside and during a pandemic, named by 164 respectively 180 out of 1000 survey participants, in the 2018 and 2020 samples (adjusted OR = 1.31, 95% CI = 0.96–1.77, *p* = 0.087). A slight increase in contacts with GPs during the pandemic is not surprising and signifies the adherence of the population to the recommendation by the Federal Office of Public Health [27]. This recommendation is to primarily call the GP when COVID-19 is suspected. Based on experiences from other countries or previous pandemics, one might have expected a higher GP workload during the current outbreak [28–30]. This might be true for the beginning of the pandemic when substantially more patients called their GPs with questions about issues related to COVID-19. However, the initial increase in GP workload due to telephone advice was balanced by a decrease in non-urgent patient care. Regular patient care such as follow-up visits were prohibited by emergency laws between March 17th, 2020 and April 26th, 2020 [31]. To date no evidence is available to support financial loss of income of GPs during the lock down phase of the pandemic. However, the emergency laws massively affected specialists that experienced a massive drop in consultations resulting in considerably less revenue. There has even been media coverage of specialist physicians in private practice applying for unemployment insurance benefits due to the sudden drop in turnover resulting from disease control measures [32]. Our finding of a significant drop in attendance to specialists is in line with this.

Pharmacies are easily accessible (i.e. spatially widespread and have long opening hours) and offer low-threshold medical services. Therefore, it is not surprising that during the COVID-19 pandemic, resulting in unprecedented restrictions in the health care system, more people (38/1000) turned towards pharmacies for medical advice than outside a pandemic (18/1000). Further, in March 2020, safety concerns were raised regarding certain medications in the context of COVID-19, such as non-steroidal anti-inflammatory drugs, angiotensin converting enzyme inhibitors [33], angiotensin receptor blockers and other renin angiotensin aldosterone system antagonists. Together with the news that the supply of certain medication and hygiene material such as masks and disinfectant was no longer guaranteed in Switzerland [34], this may have contributed to an increase in advice-seeking in pharmacies.

In order to deal with the COVID-19 pandemic, hospitals mobilized a large part of their resources. While all other hospital departments were running low, the capacities in the 82 intensive care units in Switzerland were increased from a total of 1000 available beds in March to 1550 beds in April [35]. Contrary to expectations based on the situation in the North of Italy with overcrowded hospitals, hospitals in Switzerland were less frequented during the pandemic than before [36]; the University Hospital of Zurich, for example, reported that the number of newly admitted patients reduced by 50% in March 2020, both in inpatient and outpatient settings [37]. In our survey, we could not observe differences in the rates of hospital care during the pandemic compared to estimates from 2018. In contrast to the inpatient setting, we observed a higher rate of attendance to the accident and emergency units during the first wave of this pandemic. This might be explained by the fact that SARS-CoV-2 testing was mostly hospital based during this time. Due to shortages in test material, only hospitalized patients and patients at risk of severe disease were tested until April 21st, 2020 [38].

A surprising finding is that the national and cantonal hotlines were not contacted more frequently. The national hotline reported 85,272 COVID-19 related calls during March and April [39, 40], meaning that approximately 1% of the Swiss population called the national hotline, which is comparable to our hotline estimate of 0.6%. However, the cantons also provided hotlines which might have been used additionally to the national hotline as in the case of the Canton Valais with 10% of the inhabitants calling the cantonal hotline [39]. These hotlines also addressed more general questions such as travelling, work and wearing masks in public transport. Considering that in our survey we focused on medical advice regarding COVID-19, it is understandable that more people contacted their GP than a hotline.

The internet and its fast development elicited an information revolution of unparalleled extent [41, 42]. It all started in 1994 with the spread of personal computers (PCs) and the increased use of internet [43]. By 1997, 41% of U.S. households possessed a PC and nearly half of U.S. internet users had sought health information on the world wide web [44]. A cross-sectional study in 2004 encompassing 851 adults from the UK [45], suggested that the majority of patients favoured to ask their GP as the main source of health advice while the internet figured as the second preferred source for health information. A report in 2009 showed that three quarters of all U.S. adults utilized the web, and 60 % have searched health or medical information on the web [46, 47]. Moreover, half have accessed a website about a particular medical condition or problem. Results from a biennial, cross-sectional survey among US adults from 2011 to 2014 [48] showed that around 45% of participants reported using the internet as the first place they go for health information compared to family/friends, health care professionals, and traditional media. The statistical analysis to predict the use of the internet as a source of health information indicated that participants who were younger, had higher socio-economical status, higher internet skills and had higher education levels were more likely to report using online sources for health information. Among the thousands of medical websites, it is not easy to find reliable health information containing trustworthy and current medical news. However, during the pandemic, respondents were much more likely to consult the internet for their health problems (26/1000) than outside the pandemic (3/1000). An advantage of the internet is that its use is contact-less medical advice, thus avoiding cross-infection during the COVID-19 pandemic. In the UK, remote assessment of COVID-19 in primary care was suggested [11]. The management of diseases in a remote or non-face-to-face method is not yet widely implemented despite its feasibility in a variety of settings, flexibility of application, and facilitative effect in delivering timely speciality advice [49].

### Strengths, limitations & generalizability

To our knowledge, this is the first study using the ecology of care framework to assess the use of health care in the general population in the context of a global pandemic. We used a representative sample to put the impact of the current health emergency into the perspective of the general population. Of course, there are limitations to the methods we used in this analysis. Most importantly, the study might be subject to several selection biases: first, people with chronic illnesses, especially those with risk factors for severe COVID-19, might be more likely to respond. Second, due to disease control measures imposed by the Swiss confederation, an online survey was carried out instead of a telephone survey, as in 2018. This explains some of the differences in socio-demographic (household size, rural vs. urban area of residence, and employment status) and digital-affinity variables such as consulting the internet for medical advice between the two samples shown in Table 1, and limits the validity of the comparison. In this context, it was not possible to include individuals over 80 years in the 2020 survey because the LINK panel did not have a representative sample of this age group for online surveys. This is unfortunate given the elevated risk among individuals over 80 years to have health problems and seek medical advice. Thus, we could not capture the impact of the burden of illness within this age group. According to the data from the Swiss Federal Statistical Office [3], 68% of deaths in the first wave in mid-April occurred in people aged 80 years and older. The peak of the first wave of the COVID-19 pandemic resulted in more than 532 (46%) additional deaths within a week in Switzerland compared to a normal mortality rate calculated based on the average of the past 5 years. However, this was only the case in persons aged over 65 years. COVID-19 had no statistically significant effect on mortality rates of younger age groups. Primary care physicians are therefore in the position to protect people who are weakened, either through age or previous illnesses, by not sending them unnecessarily to hospitals or emergency rooms.

Further, the comparison of low rates in health care utilisation, in particular of in-patient hospital care, or subgroup analysis within the Italian speaking region, have to be interpreted with caution due to limited power of such tests. The results are generalisable to the language-assimilated, Swiss population aged 18 to 80 years, but not to other populations within different healthcare systems, and to paediatric populations.

## Conclusion

Compared to 2018, GPs remained the most important source of medical advice for the population during the first wave of the COVID-19 pandemic. While the estimates of health problems and the need for medical advice remained constant, individuals seemed to change their provider choice during the pandemic with an increased utilisation of pharmacies and accident and emergency departments, which represent easily accessible and low-threshold medical services.

## Supplementary Information

**Additional file 1: File 1.** Cumulative sum of COVID-19 cases in Switzerland. **File 2.** Questionnaire items. **File3.** Multivariable logistic regression model of the association of demographic variables on medical ecology in terms of health problems, medical advice seeking and hospitalisation. **File 4.** Medical ecology in terms of type of care according to age group relative to all respondents in the 2020 survey (*n* = 1022). We present items of health care use which differed across age groups. **File 5.** Medical ecology in terms of type of care according to language region relative to all respondents in the 2020 survey (*n* = 1022). We present items of health care use which differed across language regions. **File 6.** Medical ecology in terms of type of care according to sex relative to all respondents in the 2020 survey (*n* = 1022). We present items of health care use which differed across men and women. **File 7.** Medical ecology in terms of type of care according to residence type relative to all respondents in the 2020 survey (*n* = 1022). We present items of health care use which differed across urban and rural residence. **File 8.** Medical ecology in terms of type of care according to employment status relative to all respondents in the 2020 survey (*n* = 1022). We present items of health care use which differed across employed and unemployed people. **File 9.** Medical ecology in terms of type of care according to household size relative to all respondents in the 2020 survey (*n* = 1022). We present items of health care use which differed across household size of 1, 2, or 3 or more people.

## Data Availability

The datasets used and/or analysed during the current study are available from the corresponding author on reasonable request.

## Abbreviations

GP: General practitioner
COVID-19: Coronavirus disease 2019
SARS-CoV-2: Coronavirus-2
