# Supplementary material for: The use of health care during the SARS-CoV-2 pandemic: repeated cross-sectional survey of the adult Swiss general population
Source: BMC Public Health. 2021 May 3;21:853. doi: 10.1186/s12889-021-10854-1 (PMC8091147; doi:10.1186/s12889-021-10854-1)
Supplement: Supplementary file 1 — Additional file 1: File 1. Cumulative sum of COVID-19 cases in Switzerland. File 2. Questionnaire items. File3. Multivariable logistic regression model of the association of demographic variables on medical ecology in terms of health problems, medical advice seeking and hospitalisation. File 4. Medical ecology in terms of type of care according to age group relative to all respondents in the 2020 survey (n = 1022). We present items of health care use which differed across age groups. File 5. Medical ecology in terms of type of care according to language region relative to all respondents in the 2020 survey (n = 1022). We present items of health care use which differed across language regions. File 6. Medical ecology in terms of type of care according to sex relative to all respondents in the 2020 survey (n = 1022). We present items of health care use which differed across men and women. File 7. Medical ecology in terms of type of care according to residence type relative to all respondents in the 2020 survey (n = 1022). We present items of health care use which differed across urban and rural residence. File 8. Medical ecology in terms of type of care according to employment status relative to all respondents in the 2020 survey (n = 1022). We present items of health care use which differed across employed and unemployed people. File 9. Medical ecology in terms of type of care according to household size relative to all respondents in the 2020 survey (n = 1022). We present items of health care use which differed across household size of 1, 2, or 3 or more people. [file 12889_2021_10854_MOESM1_ESM.docx]

# Supplementary material

**File 1: Cumulative sum of COVID-19 cases in Switzerland**


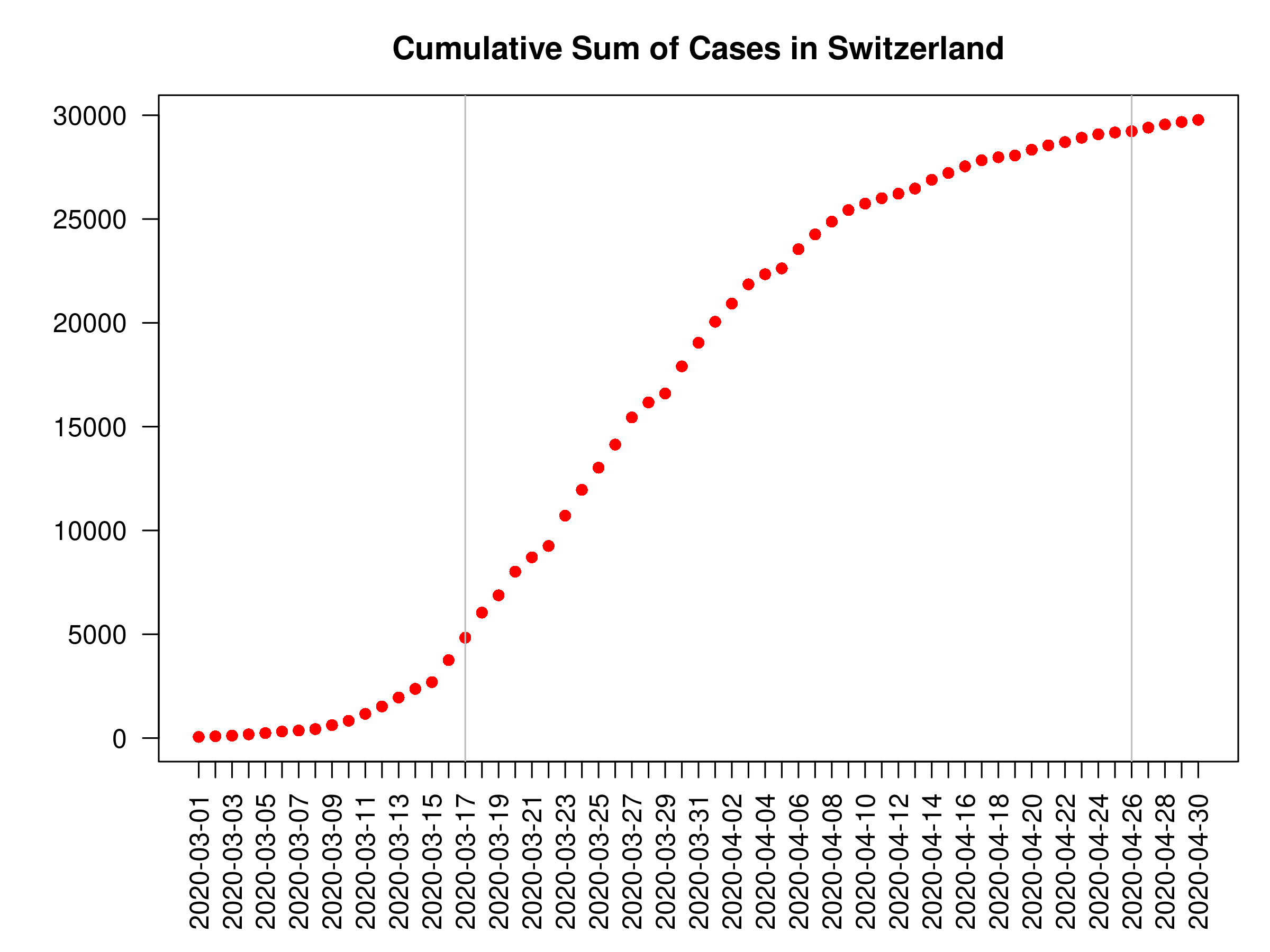


The vertical grey lines indicate the lockdown period from 17^th^ of March to 26^th^ of April

### File 2: Questionnaire items

- Did you have any health problems in the past 8 weeks? This includes serious illnesses but also minor illnesses, e.g. Headache, a cold or hay fever.
  - Did you have one or more health problems?
  - Have you sought medical advice about this health problem / problems?
    - … internet
    - … pharmacy
    - … drugstore
    - … telephone medical advice center
    - … general practitioner
    - … specialist physician
    - … accident and emergency unit
    - … outpatient clinic
    - … physiotherapy
    - … dentist
    - … Complementary and alternative medicine ppractitioner
    - … family/friends
    - … psychotherapist or psychologist
    - … hotline BAG
    - … others
  - Did you have an inpatient hospital stay due to your health problem (s) in the past 8 weeks, i.e. with an overnight stay?
    - Have you been in normal care unit or in intensive care unit?
    - Did you need mechanic ventilation?
    - Did you have an operation?
    - Have you been in regional hospital, university hospital, cantonal hospital, private hospital?
    - Did you go to rehabilitation after your hospital stay?
    - Did you have support from ambulatory nursing care after your discharge from hospital?
- Are you permanently registered with a general practitioner?
  - Did you need to contact your GP due to COVID-19?
  - How did you contact your GP? (by phone, e-mail, WhatsApp/texting, going to the practice in person)

### File 3: Multivariable logistic regression model of the association of demographic variables on medical ecology in terms of health problems, medical advice seeking and hospitalisation.

| … had 1 or more health problems | OR | Lower CI | Upper CI | Pr(>\|t\|) |
| --- | --- | --- | --- | --- |
| Household size | 0.76 | 0.65 | 0.9 | 0.001 |
| Urban vs. rural | 1.42 | 1.11 | 1.82 | 0.006 |
| Not employed vs. employed | 1.2 | 0.94 | 1.52 | 0.137 |
| Men vs. Women | 0.65 | 0.53 | 0.8 | <0.001 |
| Age (per 10 years) | 0.8 | 0.74 | 0.86 | <0.001 |
| Italian vs. German-speaking | 0.87 | 0.54 | 1.42 | 0.583 |
| French vs. German-speaking | 0.81 | 0.64 | 1.02 | 0.073 |
| Survey 2020 vs. 2018 | 0.85 | 0.69 | 1.04 | 0.106 |
| … asked for medical advice |  |  |  |  |
| Household size | 0.9 | 0.73 | 1.11 | 0.322 |
| Urban vs. rural | 1.01 | 0.72 | 1.42 | 0.941 |
| Not employed vs. employed | 1.11 | 0.8 | 1.53 | 0.538 |
| Men vs. Women | 0.82 | 0.61 | 1.1 | 0.187 |
| Age (per 10 years) | 1.2 | 1.08 | 1.33 | <0.001 |
| Italian  vs. German-speaking | 1.32 | 0.67 | 2.59 | 0.427 |
| French vs. German-speaking | 1.63 | 1.15 | 2.32 | 0.006 |
| Survey 2020 vs. 2018 | 1.01 | 0.76 | 1.34 | 0.95 |
| Type of medical advice |  |  |  |  |
| … internet |  |  |  |  |
| Household size | 0.79 | 0.49 | 1.27 | 0.322 |
| Urban vs. rural | 1.43 | 0.46 | 4.48 | 0.535 |
| Not employed vs. employed | 1.74 | 0.71 | 4.31 | 0.229 |
| Men vs. Women | 1.84 | 0.85 | 3.97 | 0.120 |
| Age (per 10 years) | 0.66 | 0.49 | 0.87 | 0.004 |
| Italian  vs. German-speaking | 0 | 0 | 0 | <0.001 |
| French vs. German-speaking | 0.85 | 0.34 | 2.15 | 0.737 |
| Survey 2020 vs. 2018 | 9.8 | 2.9 | 33.09 | <0.001 |
| … pharmacy |  |  |  |  |
| Household size | 0.61 | 0.38 | 0.96 | 0.034 |
| Urban vs. rural | 1.2 | 0.54 | 2.7 | 0.654 |
| Not employed vs. employed | 0.29 | 0.1 | 0.81 | 0.019 |
| Men vs. Women | 0.82 | 0.37 | 1.81 | 0.627 |
| Age (per 10 years) | 0.79 | 0.62 | 0.99 | 0.040 |
| Italian  vs. German-speaking | 0.37 | 0.05 | 2.89 | 0.343 |
| French vs. German-speaking | 0.99 | 0.42 | 2.3 | 0.974 |
| Survey 2020 vs. 2018 | 2.64 | 1.08 | 6.45 | 0.034 |
| … drugstore |  |  |  |  |
| Household size | 0.81 | 0.39 | 1.69 | 0.569 |
| Urban vs. rural | 0.53 | 0.11 | 2.69 | 0.447 |
| Not employed vs. employed | 0.5 | 0.06 | 4.27 | 0.527 |
| Men vs. Women | 2.21 | 0.46 | 10.56 | 0.321 |
| Age (per 10 years) | 0.83 | 0.52 | 1.34 | 0.456 |
| Italian  vs. German-speaking | 0 | 0 | 0 | <0.001 |
| French vs. German-speaking | 0.46 | 0.05 | 4.19 | 0.491 |
| Survey 2020 vs. 2018 | 2.82 | 0.4 | 19.74 | 0.297 |
| … telephone medical advice center |  |  |  |  |
| Household size | 0.68 | 0.28 | 1.67 | 0.4 |
| Urban vs. rural | 4.53 | 0.59 | 34.93 | 0.148 |
| Not employed vs. employed | 3.14 | 0.95 | 10.4 | 0.062 |
| Men vs. Women | 1.46 | 0.42 | 5.07 | 0.554 |
| Age (per 10 years) | 0.8 | 0.55 | 1.17 | 0.256 |
| Italian  vs. German-speaking | 0 | 0 | 0 | <0.001 |
| French vs. German-speaking | 0.63 | 0.13 | 3 | 0.561 |
| Survey 2020 vs. 2018 | 1.87 | 0.63 | 5.54 | 0.259 |
| … general practitioner |  |  |  |  |
| Household size | 0.99 | 0.72 | 1.37 | 0.963 |
| Urban vs. rural | 0.96 | 0.54 | 1.68 | 0.879 |
| Not employed vs. employed | 0.81 | 0.48 | 1.39 | 0.448 |
| Men vs. Women | 0.98 | 0.6 | 1.58 | 0.921 |
| Age (per 10 years) | 1.16 | 1 | 1.35 | 0.049 |
| Italian  vs. German-speaking | 2.1 | 0.4 | 10.93 | 0.377 |
| French vs. German-speaking | 0.77 | 0.44 | 1.35 | 0.369 |
| Survey 2020 vs. 2018 | 0.96 | 0.61 | 1.52 | 0.877 |
| … specialist physician |  |  |  |  |
| Household size | 1.03 | 0.73 | 1.44 | 0.887 |
| Urban vs. rural | 0.64 | 0.36 | 1.15 | 0.136 |
| Not employed vs. employed | 0.76 | 0.4 | 1.44 | 0.404 |
| Men vs. Women | 1.33 | 0.79 | 2.24 | 0.281 |
| Age (per 10 years) | 1.25 | 1.05 | 1.47 | 0.011 |
| Italian  vs. German-speaking | 2.84 | 1.06 | 7.6 | 0.039 |
| French vs. German-speaking | 1.31 | 0.71 | 2.39 | 0.388 |
| Survey 2020 vs. 2018 | 0.46 | 0.28 | 0.75 | 0.002 |
| … accident and emergency unit |  |  |  |  |
| Household size | 0.97 | 0.64 | 1.47 | 0.885 |
| Urban vs. rural | 1.31 | 0.51 | 3.36 | 0.574 |
| Not employed vs. employed | 1 | 0.54 | 1.87 | 0.994 |
| Men vs. Women | 1.13 | 0.57 | 2.28 | 0.723 |
| Age (per 10 years) | 1.09 | 0.83 | 1.44 | 0.527 |
| Italian  vs. German-speaking | 0.58 | 0.07 | 4.46 | 0.599 |
| French vs. German-speaking | 2.03 | 0.94 | 4.39 | 0.073 |
| Survey 2020 vs. 2018 | 2.54 | 1.19 | 5.44 | 0.017 |
| … outpatient clinic |  |  |  |  |
| Household size | 1.18 | 0.7 | 1.99 | 0.526 |
| Urban vs. rural | 1.12 | 0.45 | 2.79 | 0.811 |
| Not employed vs. employed | 0.92 | 0.34 | 2.52 | 0.869 |
| Men vs. Women | 2.06 | 0.94 | 4.5 | 0.07 |
| Age (per 10 years) | 1.52 | 1.18 | 1.95 | 0.001 |
| Italian  vs. German-speaking | 0.88 | 0.17 | 4.49 | 0.881 |
| French vs. German-speaking | 1.4 | 0.58 | 3.38 | 0.449 |
| Survey 2020 vs. 2018 | 0.91 | 0.41 | 2 | 0.805 |
| … physiotherapy |  |  |  |  |
| Household size | 2.05 | 0.87 | 4.86 | 0.103 |
| Urban vs. rural | 0.54 | 0.19 | 1.51 | 0.24 |
| Not employed vs. employed | 0.94 | 0.23 | 3.83 | 0.928 |
| Men vs. Women | 1.77 | 0.58 | 5.42 | 0.318 |
| Age (per 10 years) | 1.16 | 0.74 | 1.83 | 0.525 |
| Italian  vs. German-speaking | 0 | 0 | 0 | <0.001 |
| French vs. German-speaking | 1.61 | 0.55 | 4.74 | 0.386 |
| Survey 2020 vs. 2018 | 4.43 | 1.11 | 17.72 | 0.036 |
| … dentist |  |  |  |  |
| Household size | 1.03 | 0.47 | 2.29 | 0.932 |
| Urban vs. rural | 0.5 | 0.09 | 2.66 | 0.418 |
| Not employed vs. employed | 2.56 | 0.66 | 9.9 | 0.174 |
| Men vs. Women | 0.72 | 0.18 | 2.93 | 0.65 |
| Age (per 10 years) | 0.85 | 0.53 | 1.39 | 0.524 |
| Italian  vs. German-speaking | 2.07 | 0.21 | 19.99 | 0.53 |
| French vs. German-speaking | 0.28 | 0.03 | 2.33 | 0.241 |
| Survey 2020 vs. 2018 | 0.96 | 0.26 | 3.57 | 0.947 |
| … complementary and alternative medicine practitioner |  |  |  |  |
| Household size | 1.37 | 0.78 | 2.39 | 0.272 |
| Urban vs. rural | 0.87 | 0.23 | 3.32 | 0.834 |
| Not employed vs. employed | 0.14 | 0.02 | 0.97 | 0.047 |
| Men vs. Women | 0.18 | 0.04 | 0.72 | 0.016 |
| Age (per 10 years) | 1.13 | 0.81 | 1.57 | 0.464 |
| Italian  vs. German-speaking | 0 | 0 | 0 | <0.001 |
| French vs. German-speaking | 0.65 | 0.16 | 2.68 | 0.547 |
| Survey 2020 vs. 2018 | 1.53 | 0.48 | 4.93 | 0.475 |
| … family/friends |  |  |  |  |
| Household size | 1.13 | 0.68 | 1.87 | 0.645 |
| Urban vs. rural | 2.26 | 0.63 | 8.08 | 0.211 |
| Not employed vs. employed | 0.68 | 0.2 | 2.26 | 0.527 |
| Men vs. Women | 0.52 | 0.19 | 1.44 | 0.207 |
| Age (per 10 years) | 0.75 | 0.54 | 1.04 | 0.086 |
| Italian  vs. German-speaking | 0 | 0 | 0 | <0.001 |
| French vs. German-speaking | 0.3 | 0.08 | 1.1 | 0.069 |
| Survey 2020 vs. 2018 | 2.15 | 0.72 | 6.39 | 0.171 |
| … psychotherapist or psychologist |  |  |  |  |
| Household size | 0.73 | 0.34 | 1.55 | 0.414 |
| Urban vs. rural | 29004659 | 13944674 | 60329143 | <0.001 |
| Not employed vs. employed | 1.69 | 0.48 | 6.01 | 0.417 |
| Men vs. Women | 0.26 | 0.07 | 0.94 | 0.041 |
| Age (per 10 years) | 0.71 | 0.48 | 1.04 | 0.079 |
| Italian  vs. German-speaking | 2.64 | 0.26 | 26.72 | 0.413 |
| French vs. German-speaking | 0.15 | 0.02 | 1.24 | 0.08 |
| … hotline BAG/cantonal hotline |  |  |  |  |
| Household size | 0.63 | 0.22 | 1.82 | 0.39 |
| Urban vs. rural | 11837936 | 2679235 | 52304752 | <0.001 |
| Not employed vs. employed | 0.57 | 0.06 | 5.01 | 0.611 |
| Men vs. Women | 0.33 | 0.03 | 3.08 | 0.329 |
| Age (per 10 years) | 0.69 | 0.41 | 1.15 | 0.157 |
| Italian  vs. German-speaking | 0 | 0 | 0 | <0.001 |
| French vs. German-speaking | 16.34 | 1.73 | 154.74 | 0.016 |
| … had in-patient hospital care … |  |  |  |  |
| Household size | 1.22 | 0.74 | 2.01 | 0.435 |
| Urban vs. rural | 1.44 | 0.61 | 3.4 | 0.405 |
| Not employed vs. employed | 3.21 | 1.65 | 6.23 | 0.001 |
| Men vs. Women | 1.6 | 0.8 | 3.18 | 0.183 |
| Age (per 10 years) | 1.18 | 0.93 | 1.51 | 0.172 |
| Italian  vs. German-speaking | 1.37 | 0.32 | 5.86 | 0.673 |
| French vs. German-speaking | 1.47 | 0.66 | 3.27 | 0.342 |
| Survey 2020 vs. 2018 | 1.45 | 0.71 | 2.95 | 0.311 |

OR = Odds ratio, CI = 95% confidence interval.

## Subgroup analysis

### File 4: Medical ecology in terms of type of care according to age group relative to all respondents in the 2020 survey (n=1022). We present items of health care use which differed across age groups.

| … had 1 or more health problems | Yes | No | % yes | Statistic* | df | p-value |
| --- | --- | --- | --- | --- | --- | --- |
| 18-29 years old | 138 | 75 | 64.78 | 8.29 | 3 | <0.001 |
| 30-44 years old | 151 | 147 | 50.6 |  |  |  |
| 45-59 years old | 151 | 155 | 49.48 |  |  |  |
| 60-79 years old | 84 | 122 | 40.83 |  |  |  |
| .. number of health problems | one | multiple | % one | Statistic* | df | p-value |
| 18-29 years old | 87 | 50 | 63.43 | 0.26 | 3 | 0.855 |
| 30-44 years old | 92 | 57 | 61.67 |  |  |  |
| 45-59 years old | 97 | 53 | 64.87 |  |  |  |
| 60-79 years old | 56 | 28 | 67.15 |  |  |  |
| … asked for medical advice | Yes | No | % yes | Statistic* | df | p-value |
| 18-29 years old | 45 | 92 | 32.88 | 5.77 | 3 | 0.001 |
| 30-44 years old | 52 | 97 | 34.86 |  |  |  |
| 45-59 years old | 75 | 75 | 49.98 |  |  |  |
| 60-79 years old | 46 | 38 | 54.82 |  |  |  |
| … internet | Yes | No | % yes | Statistic* | df | p-value |
| 18-29 years old | 12 | 33 | 25.89 | 3.93 | 3 | 0.008 |
| 30-44 years old | 5 | 47 | 9.45 |  |  |  |
| 45-59 years old | 4 | 71 | 5.21 |  |  |  |
| 60-79 years old | 6 | 40 | 13.43 |  |  |  |
| … pharmacy | Yes | No | % yes | Statistic* | df | p-value |
| 18-29 years old | 11 | 34 | 23.99 | 1.24 | 3 | 0.295 |
| 30-44 years old | 12 | 40 | 22.53 |  |  |  |
| 45-59 years old | 11 | 64 | 14.72 |  |  |  |
| 60-79 years old | 5 | 41 | 11.43 |  |  |  |
| … drugstore | Yes | No | % yes | Statistic* | df | p-value |
| 18-29 years old | 2 | 43 | 4.4 | 0.41 | 3 | 0.743 |
| 30-44 years old | 2 | 50 | 3.76 |  |  |  |
| 45-59 years old | 1 | 74 | 1.35 |  |  |  |
| 60-79 years old | 1 | 45 | 2.26 |  |  |  |
| … telephone medical advice center | Yes | No | % yes | Statistic* | df | p-value |
| 18-29 years old | 2 | 43 | 4.31 | 2.95 | 3 | 0.032 |
| 30-44 years old | 6 | 46 | 11.36 |  |  |  |
| 45-59 years old | 0 | 75 | 0 |  |  |  |
| 60-79 years old | 3 | 43 | 6.7 |  |  |  |
| … general practitioner | Yes | No | % yes | Statistic* | df | p-value |
| 18-29 years old | 23 | 22 | 52.16 | 2.5 | 3 | 0.058 |
| 30-44 years old | 34 | 19 | 64.24 |  |  |  |
| 45-59 years old | 57 | 18 | 75.66 |  |  |  |
| 60-79 years old | 33 | 13 | 70.86 |  |  |  |
| … specialist physician | Yes | No | % yes | Statistic* | df | p-value |
| 18-29 years old | 7 | 38 | 15.41 | 0.72 | 3 | 0.54 |
| 30-44 years old | 8 | 44 | 15.18 |  |  |  |
| 45-59 years old | 15 | 60 | 20.46 |  |  |  |
| 60-79 years old | 12 | 34 | 25.45 |  |  |  |
| … accident and emergency unit | Yes | No | % yes | Statistic* | df | p-value |
| 18-29 years old | 6 | 39 | 12.99 | 0.37 | 3 | 0.774 |
| 30-44 years old | 4 | 48 | 7.75 |  |  |  |
| 45-59 years old | 10 | 65 | 13.25 |  |  |  |
| 60-79 years old | 6 | 40 | 13.17 |  |  |  |
| … outpatient clinic | Yes | No | % yes | Statistic* | df | p-value |
| 18-29 years old | 0 | 45 | 0 | 2.98 | 3 | 0.03 |
| 30-44 years old | 2 | 50 | 3.85 |  |  |  |
| 45-59 years old | 9 | 66 | 12.24 |  |  |  |
| 60-79 years old | 6 | 40 | 13.78 |  |  |  |
| … physiotherapy | Yes | No | % yes | Statistic* | df | p-value |
| 18-29 years old | 3 | 42 | 6.45 | 1.5 | 3 | 0.212 |
| 30-44 years old | 1 | 51 | 1.96 |  |  |  |
| 45-59 years old | 7 | 68 | 9.47 |  |  |  |
| 60-79 years old | 1 | 45 | 2.22 |  |  |  |
| … dentist | Yes | No | % yes | Statistic* | df | p-value |
| 18-29 years old | 3 | 42 | 6.64 | 2.02 | 3 | 0.108 |
| 30-44 years old | 0 | 52 | 0 |  |  |  |
| 45-59 years old | 2 | 73 | 2.74 |  |  |  |
| 60-79 years old | 0 | 46 | 0 |  |  |  |
| … complementary and alternative medicine practitioner | Yes | No | % yes | Statistic* | df | p-value |
| 18-29 years old | 1 | 44 | 2.18 | 0.42 | 3 | 0.736 |
| 30-44 years old | 3 | 49 | 5.65 |  |  |  |
| 45-59 years old | 5 | 70 | 6.68 |  |  |  |
| 60-79 years old | 3 | 43 | 6.6 |  |  |  |
| … family/friends | Yes | No | % yes | Statistic* | df | p-value |
| 18-29 years old | 8 | 37 | 17.36 | 4.44 | 3 | 0.004 |
| 30-44 years old | 2 | 50 | 3.75 |  |  |  |
| 45-59 years old | 4 | 71 | 5.4 |  |  |  |
| 60-79 years old | 0 | 46 | 0 |  |  |  |
| … others(associate of medhome, atlasoid, chiropractor) | Yes | No | % yes | Statistic* | df | p-value |
| 18-29 years old | 2 | 43 | 4.37 | 1.03 | 3 | 0.377 |
| 30-44 years old | 1 | 51 | 1.96 |  |  |  |
| 45-59 years old | 0 | 75 | 0 |  |  |  |
| 60-79 years old | 1 | 45 | 2.12 |  |  |  |
| … No Answer | Yes | No | % yes | Statistic* | df | p-value |
| 18-29 years old | 0 | 45 | 0 | 1.35 | 3 | 0.255 |
| 30-44 years old | 0 | 52 | 0 |  |  |  |
| 45-59 years old | 0 | 75 | 0 |  |  |  |
| 60-79 years old | 1 | 45 | 2.41 |  |  |  |
| psychotherapist or psychologist | Yes | No | % yes | Statistic* | df | p-value |
| 18-29 years old | 3 | 42 | 6.64 | 1.7 | 3 | 0.166 |
| 30-44 years old | 7 | 45 | 13.27 |  |  |  |
| 45-59 years old | 4 | 71 | 5.37 |  |  |  |
| 60-79 years old | 1 | 45 | 2.26 |  |  |  |
| … hotline BAG | Yes | No | % yes | Statistic* | df | p-value |
| 18-29 years old | 1 | 44 | 2.18 | 0.68 | 3 | 0.566 |
| 30-44 years old | 2 | 50 | 3.85 |  |  |  |
| 45-59 years old | 3 | 72 | 3.94 |  |  |  |
| 60-79 years old | 0 | 46 | 0 |  |  |  |
| … had in-patient hospital care | Yes | No | % yes | Statistic* | df | p-value |
| 18-29 years old | 5 | 132 | 3.58 | 4.44 | 3 | 0.004 |
| 30-44 years old | 1 | 149 | 0.65 |  |  |  |
| 45-59 years old | 5 | 145 | 3.38 |  |  |  |
| 60-79 years old | 8 | 76 | 10.06 |  |  |  |
| … in normal care unit or ICU? | ICU | Normal care | % ICU | Statistic* | df | p-value |
| 18-29 years old | 0 | 5 | 0 | 3.45 | 3 | 0.016 |
| 30-44 years old | 1 | 0 | 100 |  |  |  |
| 45-59 years old | 0 | 5 | 0 |  |  |  |
| 60-79 years old | 1 | 7 | 12.11 |  |  |  |
| … had a surgical procedure | Yes | No | % yes | Statistic* | df | p-value |
| 18-29 years old | 1 | 4 | 20.84 | 0.91 | 3 | 0.435 |
| 30-44 years old | 0 | 1 | 0 |  |  |  |
| 45-59 years old | 2 | 3 | 40.48 |  |  |  |
| 60-79 years old | 5 | 3 | 60.19 |  |  |  |
| … were in rehabilitation clinic | Yes | No | % yes | Statistic* | df | p-value |
| 18-29 years old | 2 | 3 | 40.4 | 0.33 | 3 | 0.8 |
| 30-44 years old | 0 | 1 | 0 |  |  |  |
| 45-59 years old | 1 | 4 | 20.52 |  |  |  |
| 60-79 years old | 2 | 6 | 23.14 |  |  |  |
| … had ambulatory nursing care | Yes | No | % yes | Statistic* | df | p-value |
| 18-29 years old | 0 | 5 | 0 | 0.41 | 3 | 0.749 |
| 30-44 years old | 0 | 1 | 0 |  |  |  |
| 45-59 years old | 1 | 4 | 19.96 |  |  |  |
| 60-79 years old | 1 | 7 | 12.11 |  |  |  |

*The chisquared test of independence was performed with the Rao-Scott second-order correction. The p-values are computed with a Satterthwaite approximation to the distribution and with denominator degrees of freedom as recommended by Thomas and Rao (1990).

### File 5: Medical ecology in terms of type of care according to language region relative to all respondents in the 2020 survey (n=1022). We present items of health care use which differed across language regions. All tables can be found in the supplementary material

| … had 1 or more health problems | Yes | No | % yes | Statistic* | df | p-value |
| --- | --- | --- | --- | --- | --- | --- |
| German-speaking | 382 | 353 | 51.99 | 1.4 | 2 | 0.247 |
| Italian-speaking | 16 | 25 | 38.63 |  |  |  |
| French-speaking | 125 | 120 | 51.16 |  |  |  |
| .. number of health problems | one | multiple | % one | Statistic* | df | p-value |
| German-speaking | 239 | 140 | 63.09 | 0.62 | 2 | 0.537 |
| Italian-speaking | 12 | 4 | 76.34 |  |  |  |
| French-speaking | 81 | 44 | 64.92 |  |  |  |
| … asked for medical advice | Yes | No | % yes | Statistic* | df | p-value |
| German-speaking | 156 | 224 | 41.03 | 0.58 | 2 | 0.562 |
| Italian-speaking | 9 | 7 | 54.08 |  |  |  |
| French-speaking | 54 | 71 | 43.1 |  |  |  |
| … internet | Yes | No | % yes | Statistic* | df | p-value |
| German-speaking | 20 | 136 | 12.68 | 0.61 | 2 | 0.542 |
| Italian-speaking | 0 | 9 | 0 |  |  |  |
| French-speaking | 7 | 47 | 12.83 |  |  |  |
| … pharmacy | Yes | No | % yes | Statistic* | df | p-value |
| German-speaking | 27 | 129 | 17.27 | 0.34 | 2 | 0.711 |
| Italian-speaking | 1 | 8 | 10.21 |  |  |  |
| French-speaking | 11 | 43 | 20.56 |  |  |  |
| … drugstore | Yes | No | % yes | Statistic* | df | p-value |
| German-speaking | 5 | 151 | 3.19 | 0.25 | 2 | 0.78 |
| Italian-speaking | 0 | 9 | 0 |  |  |  |
| French-speaking | 1 | 53 | 1.89 |  |  |  |
| … telephone medical advice center | Yes | No | % yes | Statistic* | df | p-value |
| German-speaking | 10 | 146 | 6.42 | 1.14 | 2 | 0.321 |
| Italian-speaking | 0 | 9 | 0 |  |  |  |
| French-speaking | 1 | 53 | 1.79 |  |  |  |
| … general practitioner | Yes | No | % yes | Statistic* | df | p-value |
| German-speaking | 108 | 48 | 69.24 | 2.47 | 2 | 0.085 |
| Italian-speaking | 8 | 1 | 89.79 |  |  |  |
| French-speaking | 31 | 23 | 57.24 |  |  |  |
| … specialist physician | Yes | No | % yes | Statistic* | df | p-value |
| German-speaking | 30 | 125 | 19.56 | 0.04 | 2 | 0.963 |
| Italian-speaking | 1 | 7 | 15.88 |  |  |  |
| French-speaking | 10 | 44 | 18.72 |  |  |  |
| … accident and emergency unit | Yes | No | % yes | Statistic* | df | p-value |
| German-speaking | 14 | 142 | 9.07 | 2.46 | 2 | 0.086 |
| Italian-speaking | 1 | 8 | 10.42 |  |  |  |
| French-speaking | 11 | 43 | 20.14 |  |  |  |
| … outpatient clinic | Yes | No | % yes | Statistic* | df | p-value |
| German-speaking | 10 | 145 | 6.58 | 0.78 | 2 | 0.455 |
| Italian-speaking | 1 | 7 | 15.88 |  |  |  |
| French-speaking | 6 | 48 | 10.99 |  |  |  |
| … physiotherapy | Yes | No | % yes | Statistic* | df | p-value |
| German-speaking | 8 | 148 | 5.22 | 0.42 | 2 | 0.658 |
| Italian-speaking | 0 | 9 | 0 |  |  |  |
| French-speaking | 4 | 50 | 7.28 |  |  |  |
| … dentist | Yes | No | % yes | Statistic* | df | p-value |
| German-speaking | 3 | 153 | 1.95 | 1.81 | 2 | 0.164 |
| Italian-speaking | 1 | 8 | 11.91 |  |  |  |
| French-speaking | 1 | 53 | 1.82 |  |  |  |
| … complementary and alternative medicine practitioner | Yes | No | % yes | Statistic* | df | p-value |
| German-speaking | 10 | 146 | 6.45 | 0.57 | 2 | 0.564 |
| Italian-speaking | 0 | 9 | 0 |  |  |  |
| French-speaking | 2 | 52 | 3.6 |  |  |  |
| … family/friends | Yes | No | % yes | Statistic* | df | p-value |
| German-speaking | 13 | 143 | 8.25 | 1.69 | 2 | 0.185 |
| Italian-speaking | 0 | 9 | 0 |  |  |  |
| French-speaking | 1 | 53 | 1.82 |  |  |  |
| … others(associate of medhome, atlasoid, chiropractor) | Yes | No | % yes | Statistic* | df | p-value |
| German-speaking | 1 | 155 | 0.66 | 2.63 | 2 | 0.072 |
| Italian-speaking | 0 | 9 | 0 |  |  |  |
| French-speaking | 3 | 51 | 5.44 |  |  |  |
| … No Answer | Yes | No | % yes | Statistic* | df | p-value |
| German-speaking | 0 | 156 | 0 | 1.54 | 2 | 0.214 |
| Italian-speaking | 0 | 9 | 0 |  |  |  |
| French-speaking | 1 | 53 | 2.05 |  |  |  |
| psychotherapist or psychologist | Yes | No | % yes | Statistic* | df | p-value |
| German-speaking | 13 | 143 | 8.35 | 1.53 | 2 | 0.217 |
| Italian-speaking | 1 | 8 | 11.91 |  |  |  |
| French-speaking | 1 | 53 | 1.79 |  |  |  |
| … hotline BAG | Yes | No | % yes | Statistic* | df | p-value |
| German-speaking | 1 | 155 | 0.63 | 5.64 | 2 | 0.004 |
| Italian-speaking | 0 | 9 | 0 |  |  |  |
| French-speaking | 5 | 49 | 9.17 |  |  |  |
| … had in-patient hospital care | Yes | No | % yes | Statistic* | df | p-value |
| German-speaking | 14 | 365 | 3.72 | 0.51 | 2 | 0.6 |
| Italian-speaking | 1 | 15 | 8.59 |  |  |  |
| French-speaking | 4 | 122 | 3.14 |  |  |  |
| … in normal care unit or ICU? | ICU | Normal care | % ICU | Statistic* | df | p-value |
| German-speaking | 2 | 12 | 14.19 | 0.38 | 2 | 0.68 |
| Italian-speaking | 0 | 1 | 0 |  |  |  |
| French-speaking | 0 | 4 | 0 |  |  |  |
| … had a surgical procedure | Yes | No | % yes | Statistic* | df | p-value |
| German-speaking | 6 | 8 | 43.71 | 0.52 | 2 | 0.595 |
| Italian-speaking | 0 | 1 | 0 |  |  |  |
| French-speaking | 2 | 2 | 50.7 |  |  |  |
| … were in rehabilitation clinic | Yes | No | % yes | Statistic* | df | p-value |
| German-speaking | 2 | 12 | 14.19 | 3.08 | 2 | 0.047 |
| Italian-speaking | 0 | 1 | 0 |  |  |  |
| French-speaking | 3 | 1 | 75.49 |  |  |  |
| … had ambulatory nursing care | Yes | No | % yes | Statistic* | df | p-value |
| German-speaking | 2 | 12 | 14.44 | 0.39 | 2 | 0.676 |
| Italian-speaking | 0 | 1 | 0 |  |  |  |
| French-speaking | 0 | 4 | 0 |  |  |  |

*The chisquared test of independence was performed with the Rao-Scott second-order correction. The p-values are computed with a Satterthwaite approximation to the distribution and with denominator degrees of freedom as recommended by Thomas and Rao (1990).

### File 6: Medical ecology in terms of type of care according to sex relative to all respondents in the 2020 survey (n=1022). We present items of health care use which differed across men and women. All tables can be found in the supplementary material

| … had 1 or more health problems | Yes | No | % yes | Statistic* | df | p-value |
| --- | --- | --- | --- | --- | --- | --- |
| Women | 280 | 222 | 55.76 | 7.99 | 1 | 0.005 |
| Men | 244 | 276 | 46.91 |  |  |  |
| .. number of health problems | one | multiple | % one | Statistic* | df | p-value |
| Women | 179 | 100 | 64.25 | 0.02 | 1 | 0.876 |
| Men | 154 | 88 | 63.59 |  |  |  |
| … asked for medical advice | Yes | No | % yes | Statistic* | df | p-value |
| Women | 117 | 161 | 42.08 | 0.01 | 1 | 0.939 |
| Men | 101 | 141 | 41.75 |  |  |  |
| … internet |  |  |  |  |  |  |
| Women | 13 | 104 | 11.03 | 0.34 | 1 | 0.562 |
| Men | 14 | 87 | 13.6 |  |  |  |
| … pharmacy |  |  |  |  |  |  |
| Women | 28 | 89 | 23.96 | 6.62 | 1 | 0.01 |
| Men | 11 | 90 | 10.66 |  |  |  |
| … drugstore |  |  |  |  |  |  |
| Women | 3 | 114 | 2.59 | 0.02 | 1 | 0.88 |
| Men | 3 | 98 | 2.93 |  |  |  |
| … telephone medical advice center |  |  |  |  |  |  |
| Women | 7 | 110 | 5.96 | 0.47 | 1 | 0.493 |
| Men | 4 | 97 | 3.93 |  |  |  |
| … general practitioner |  |  |  |  |  |  |
| Women | 73 | 44 | 62.46 | 2.44 | 1 | 0.119 |
| Men | 73 | 28 | 72.43 |  |  |  |
| … specialist physician |  |  |  |  |  |  |
| Women | 20 | 97 | 17.19 | 0.65 | 1 | 0.42 |
| Men | 22 | 79 | 21.55 |  |  |  |
| … accident and emergency unit |  |  |  |  |  |  |
| Women | 12 | 105 | 10.15 | 0.71 | 1 | 0.4 |
| Men | 14 | 87 | 13.85 |  |  |  |
| … outpatient clinic |  |  |  |  |  |  |
| Women | 6 | 111 | 5.13 | 2.83 | 1 | 0.093 |
| Men | 12 | 90 | 11.4 |  |  |  |
| … physiotherapy |  |  |  |  |  |  |
| Women | 5 | 112 | 4.23 | 0.82 | 1 | 0.366 |
| Men | 7 | 94 | 7.03 |  |  |  |
| … dentist |  |  |  |  |  |  |
| Women | 3 | 114 | 2.54 | 0.06 | 1 | 0.808 |
| Men | 2 | 99 | 2.04 |  |  |  |
| … complementary and alternative medicine practitioner |  |  |  |  |  |  |
| Women | 9 | 108 | 7.69 | 2.36 | 1 | 0.125 |
| Men | 3 | 98 | 2.95 |  |  |  |
| … family/friends |  |  |  |  |  |  |
| Women | 10 | 107 | 8.49 | 2.03 | 1 | 0.155 |
| Men | 4 | 97 | 3.83 |  |  |  |
| psychotherapist or psychologist |  |  |  |  |  |  |
| Women | 12 | 105 | 10.19 | 4.33 | 1 | 0.038 |
| Men | 3 | 98 | 3.01 |  |  |  |
| … hotline BAG |  |  |  |  |  |  |
| Women | 5 | 112 | 4.2 | 2.05 | 1 | 0.152 |
| Men | 1 | 100 | 1.01 |  |  |  |
| … had in-patient hospital care … | Yes | No | % yes | Statistic* | df | p-value |
| Women | 6 | 273 | 2.15 | 4.08 | 1 | 0.044 |
| Men | 13 | 229 | 5.54 |  |  |  |
| … in normal care unit | ICU | Normal care | % ICU | Statistic* | df | p-value |
| Women | 0 | 6 | 0 | 1.05 | 1 | 0.305 |
| Men | 2 | 11 | 14.91 |  |  |  |
| … had a surgical procedure | Yes | No | % yes | Statistic* | df | p-value |
| Women | 2 | 4 | 34.22 | 0.21 | 1 | 0.643 |
| Men | 6 | 7 | 45.57 |  |  |  |
| … were in rehabilitation clinic |  |  |  |  |  |  |
| Women | 0 | 6 | 0 | 3.23 | 1 | 0.073 |
| Men | 5 | 8 | 37.12 |  |  |  |
| … had ambulatory nursing care |  |  |  |  |  |  |
| Women | 1 | 5 | 16.9 | 0.37 | 1 | 0.542 |
| Men | 1 | 12 | 7.63 |  |  |  |

*The chisquared test of independence was performed with the Rao-Scott second-order correction. The p-values are computed with a Satterthwaite approximation to the distribution and with denominator degrees of freedom as recommended by Thomas and Rao (1990).

### File 7: Medical ecology in terms of type of care according to residence type relative to all respondents in the 2020 survey (n=1022). We present items of health care use which differed across urban and rural residence. All tables can be found in the supplementary material

| … had 1 or more health problems | Yes | No | % yes | Statistic* | df | p-value |
| --- | --- | --- | --- | --- | --- | --- |
| Rural | 108 | 115 | 48.58 | 0.81 | 1 | 0.368 |
| Urban | 415 | 384 | 52 |  |  |  |
| .. number of health problems | One | more | % one |  |  |  |
| Rural | 71 | 36 | 66.53 | 0.39 | 1 | 0.531 |
| Urban | 262 | 152 | 63.27 |  |  |  |
| … asked for medical advice |  |  |  |  |  |  |
| Rural | 47 | 61 | 43.49 | 0.14 | 1 | 0.713 |
| Urban | 172 | 242 | 41.52 |  |  |  |
| … internet |  |  |  |  |  |  |
| Rural | 3 | 44 | 6.38 | 1.9 | 1 | 0.169 |
| Urban | 24 | 148 | 13.8 |  |  |  |
| … pharmacy |  |  |  |  |  |  |
| Rural | 8 | 39 | 17 | 0.03 | 1 | 0.871 |
| Urban | 31 | 141 | 18.02 |  |  |  |
| … drugstore |  |  |  |  |  |  |
| Rural | 2 | 45 | 4.34 | 0.56 | 1 | 0.455 |
| Urban | 4 | 168 | 2.31 |  |  |  |
| … telephone medical advice center |  |  |  |  |  |  |
| Rural | 1 | 46 | 2.11 | 1.07 | 1 | 0.302 |
| Urban | 10 | 162 | 5.81 |  |  |  |
| … general practitioner |  |  |  |  |  |  |
| Rural | 35 | 12 | 74.58 | 1.51 | 1 | 0.219 |
| Urban | 112 | 60 | 65.04 |  |  |  |
| … specialist physician |  |  |  |  |  |  |
| Rural | 15 | 32 | 31.09 | 5.26 | 1 | 0.022 |
| Urban | 27 | 144 | 15.98 |  |  |  |
| … accident and emergency unit |  |  |  |  |  |  |
| Rural | 1 | 46 | 2.23 | 5.07 | 1 | 0.025 |
| Urban | 25 | 147 | 14.48 |  |  |  |
| … outpatient clinic |  |  |  |  |  |  |
| Rural | 3 | 44 | 6.46 | 0.2 | 1 | 0.657 |
| Urban | 15 | 157 | 8.46 |  |  |  |
| … physiotherapy |  |  |  |  |  |  |
| Rural | 4 | 43 | 8.89 | 1.26 | 1 | 0.262 |
| Urban | 8 | 164 | 4.61 |  |  |  |
| … dentist |  |  |  |  |  |  |
| Rural | 1 | 46 | 2.23 | 0 | 1 | 0.969 |
| Urban | 4 | 168 | 2.33 |  |  |  |
| … complementary and alternative medicine practitioner |  |  |  |  |  |  |
| Rural | 3 | 44 | 6.54 | 0.12 | 1 | 0.725 |
| Urban | 9 | 163 | 5.21 |  |  |  |
| … family/friends |  |  |  |  |  |  |
| Rural | 1 | 46 | 2.17 | 1.71 | 1 | 0.191 |
| Urban | 13 | 159 | 7.46 |  |  |  |
| psychotherapist or psychologist |  |  |  |  |  |  |
| Rural | 0 | 47 | 0 | 4.58 | 1 | 0.033 |
| Urban | 15 | 157 | 8.73 |  |  |  |
| … hotline BAG |  |  |  |  |  |  |
| Rural | 0 | 47 | 0 | 1.71 | 1 | 0.191 |
| Urban | 6 | 166 | 3.46 |  |  |  |
| … had in-patient hospital care … |  |  |  |  |  |  |
| Rural | 1 | 106 | 0.94 | 2.87 | 1 | 0.09 |
| Urban | 18 | 395 | 4.45 |  |  |  |
| … in normal care unit | ICU | Normal care | % ICU |  |  |  |
| Rural | 0 | 1 | 0 | 0.13 | 1 | 0.723 |
| Urban | 2 | 16 | 10.87 |  |  |  |
| … had a surgical procedure |  |  |  |  |  |  |
| Rural | 0 | 1 | 0 | 0.8 | 1 | 0.373 |
| Urban | 8 | 10 | 44.38 |  |  |  |
| … were in rehabilitation clinic |  |  |  |  |  |  |
| Rural | 0 | 1 | 0 | 0.38 | 1 | 0.539 |
| Urban | 5 | 13 | 27.06 |  |  |  |
| … had ambulatory nursing care |  |  |  |  |  |  |
| Rural | 1 | 0 | 100 | 9.18 | 1 | 0.003 |
| Urban | 1 | 17 | 5.56 |  |  |  |

*The chisquared test of independence was performed with the Rao-Scott second-order correction. The p-values are computed with a Satterthwaite approximation to the distribution and with denominator degrees of freedom as recommended by Thomas and Rao (1990).

### File 8: Medical ecology in terms of type of care according to employment status relative to all respondents in the 2020 survey (n=1022). We present items of health care use which differed across employed and unemployed people.

| … had 1 or more health problems | Yes | No | % yes | Statistic* | df | p-value |
| --- | --- | --- | --- | --- | --- | --- |
| Employed | 397 | 365 | 52.07 | 0.7 | 1 | 0.402 |
| Not employed | 125 | 130 | 49.02 |  |  |  |
| .. number of health problems | One | more | % one |  |  |  |
| Employed | 257 | 137 | 65.3 | 1.57 | 1 | 0.21 |
| Not employed | 74 | 51 | 59.09 |  |  |  |
| … asked for medical advice |  |  |  |  |  |  |
| Employed | 165 | 229 | 41.95 | 0 | 1 | 0.965 |
| Not employed | 52 | 73 | 41.72 |  |  |  |
| … internet |  |  |  |  |  |  |
| Employed | 21 | 145 | 12.47 | 0.02 | 1 | 0.875 |
| Not employed | 6 | 46 | 11.65 |  |  |  |
| … pharmacy |  |  |  |  |  |  |
| Employed | 36 | 129 | 21.64 | 6.35 | 1 | 0.012 |
| Not employed | 3 | 49 | 6 |  |  |  |
| … drugstore |  |  |  |  |  |  |
| Employed | 5 | 160 | 3 | 0.15 | 1 | 0.701 |
| Not employed | 1 | 51 | 1.99 |  |  |  |
| … telephone medical advice center |  |  |  |  |  |  |
| Employed | 8 | 157 | 4.79 | 0.09 | 1 | 0.766 |
| Not employed | 3 | 49 | 5.83 |  |  |  |
| … general practitioner |  |  |  |  |  |  |
| Employed | 111 | 55 | 66.92 | 0 | 1 | 0.995 |
| Not employed | 35 | 17 | 66.96 |  |  |  |
| … specialist physician |  |  |  |  |  |  |
| Employed | 29 | 136 | 17.36 | 1.62 | 1 | 0.203 |
| Not employed | 13 | 39 | 25.43 |  |  |  |
| … accident and emergency unit |  |  |  |  |  |  |
| Employed | 22 | 143 | 13.2 | 1.05 | 1 | 0.306 |
| Not employed | 4 | 48 | 7.87 |  |  |  |
| … outpatient clinic |  |  |  |  |  |  |
| Employed | 10 | 155 | 6.13 | 3.35 | 1 | 0.067 |
| Not employed | 7 | 45 | 14.22 |  |  |  |
| … physiotherapy |  |  |  |  |  |  |
| Employed | 9 | 156 | 5.47 | 0.01 | 1 | 0.928 |
| Not employed | 3 | 49 | 5.8 |  |  |  |
| … dentist |  |  |  |  |  |  |
| Employed | 3 | 162 | 1.84 | 0.7 | 1 | 0.405 |
| Not employed | 2 | 50 | 3.84 |  |  |  |
| … complementary and alternative medicine practitioner |  |  |  |  |  |  |
| Employed | 10 | 155 | 6.05 | 0.37 | 1 | 0.542 |
| Not employed | 2 | 50 | 3.83 |  |  |  |
| … family/friends |  |  |  |  |  |  |
| Employed | 12 | 153 | 7.18 | 0.79 | 1 | 0.375 |
| Not employed | 2 | 50 | 3.76 |  |  |  |
| psychotherapist or psychologist |  |  |  |  |  |  |
| Employed | 11 | 154 | 6.67 | 0.05 | 1 | 0.816 |
| Not employed | 4 | 48 | 7.61 |  |  |  |
| … hotline BAG |  |  |  |  |  |  |
| Employed | 5 | 160 | 3.01 | 0.19 | 1 | 0.662 |
| Not employed | 1 | 51 | 1.88 |  |  |  |
| … had in-patient hospital care |  |  |  |  |  |  |
| Employed | 11 | 383 | 2.78 | 4 | 1 | 0.046 |
| Not employed | 8 | 117 | 6.75 |  |  |  |
| … in normal care unit | ICU | Normal care | % yes | Statistic* | df | p-value |
| Employed | 1 | 10 | 8.91 | 0.05 | 1 | 0.819 |
| Not employed | 1 | 7 | 12.11 |  |  |  |
| … had a surgical procedure |  |  |  |  |  |  |
| Employed | 5 | 6 | 46.34 | 0.18 | 1 | 0.668 |
| Not employed | 3 | 5 | 36.51 |  |  |  |
| … were in rehabilitation clinic |  |  |  |  |  |  |
| Employed | 3 | 8 | 27 | 0.02 | 1 | 0.877 |
| Not employed | 2 | 6 | 23.9 |  |  |  |
| … had ambulatory nursing care |  |  |  |  |  |  |
| Employed | 1 | 10 | 9.25 | 0.04 | 1 | 0.84 |
| Not employed | 1 | 7 | 12.11 |  |  |  |

*The chisquared test of independence was performed with the Rao-Scott second-order correction. The p-values are computed with a Satterthwaite approximation to the distribution and with denominator degrees of freedom as recommended by Thomas and Rao (1990).

### File 9: Medical ecology in terms of type of care according to household size relative to all respondents in the 2020 survey (n=1022). We present items of health care use which differed across household size of 1,2,or 3 or more people.

| … had 1 or more health problems | Yes | No | % yes | Statistic* | df | p-value |
| --- | --- | --- | --- | --- | --- | --- |
| 1 person | 107 | 84 | 55.97 | 1.17 | 2 | 0.312 |
| 2 persons | 179 | 185 | 49.23 |  |  |  |
| 3 or more persons | 236 | 230 | 50.69 |  |  |  |
| .. number of health problems | one | more | % one |  |  |  |
| 1 person | 66 | 40 | 62.21 | 0.1 | 2 | 0.907 |
| 2 persons | 114 | 64 | 64.15 |  |  |  |
| 3 or more persons | 151 | 83 | 64.68 |  |  |  |
| … asked for medical advice | Yes | No | % yes | Statistic* | df | p-value |
| 1 person | 48 | 58 | 45.31 | 0.92 | 2 | 0.399 |
| 2 persons | 79 | 100 | 44.07 |  |  |  |
| 3 or more persons | 91 | 143 | 38.68 |  |  |  |
| … internet |  |  |  |  |  |  |
| 1 person | 7 | 41 | 14.27 | 0.45 | 2 | 0.638 |
| 2 persons | 11 | 68 | 13.91 |  |  |  |
| 3 or more persons | 9 | 82 | 9.79 |  |  |  |
| … pharmacy |  |  |  |  |  |  |
| 1 person | 11 | 37 | 22.84 | 0.82 | 2 | 0.439 |
| 2 persons | 15 | 64 | 18.95 |  |  |  |
| 3 or more persons | 13 | 78 | 14.32 |  |  |  |
| … drugstore |  |  |  |  |  |  |
| 1 person | 1 | 47 | 2.04 | 1.3 | 2 | 0.274 |
| 2 persons | 4 | 75 | 5.09 |  |  |  |
| 3 or more persons | 1 | 90 | 1.12 |  |  |  |
| … telephone medical advice center |  |  |  |  |  |  |
| 1 person | 5 | 43 | 10.34 | 1.96 | 2 | 0.141 |
| 2 persons | 2 | 77 | 2.56 |  |  |  |
| 3 or more persons | 4 | 87 | 4.37 |  |  |  |
| … general practitioner |  |  |  |  |  |  |
| 1 person | 35 | 13 | 72.88 | 1.19 | 2 | 0.305 |
| 2 persons | 55 | 24 | 69.8 |  |  |  |
| 3 or more persons | 55 | 35 | 61.24 |  |  |  |
| … specialist physician |  |  |  |  |  |  |
| 1 person | 6 | 42 | 12.56 | 0.97 | 2 | 0.381 |
| 2 persons | 15 | 64 | 18.64 |  |  |  |
| 3 or more persons | 20 | 70 | 22.34 |  |  |  |
| … accident and emergency unit |  |  |  |  |  |  |
| 1 person | 7 | 41 | 14.26 | 0.27 | 2 | 0.766 |
| 2 persons | 8 | 71 | 10.04 |  |  |  |
| 3 or more persons | 11 | 79 | 12.31 |  |  |  |
| … outpatient clinic |  |  |  |  |  |  |
| 1 person | 4 | 44 | 8.42 | 0.09 | 2 | 0.912 |
| 2 persons | 7 | 72 | 8.93 |  |  |  |
| 3 or more persons | 6 | 84 | 7.15 |  |  |  |
| … physiotherapy |  |  |  |  |  |  |
| 1 person | 1 | 47 | 1.99 | 1.09 | 2 | 0.337 |
| 2 persons | 4 | 75 | 5.06 |  |  |  |
| 3 or more persons | 7 | 83 | 7.88 |  |  |  |
| … dentist |  |  |  |  |  |  |
| 1 person | 0 | 48 | 0 | 1.64 | 2 | 0.193 |
| 2 persons | 1 | 78 | 1.3 |  |  |  |
| 3 or more persons | 4 | 86 | 4.44 |  |  |  |
| … complementary and alternative medicine practitioner |  |  |  |  |  |  |
| 1 person | 2 | 46 | 4.1 | 0.21 | 2 | 0.809 |
| 2 persons | 5 | 74 | 6.32 |  |  |  |
| 3 or more persons | 4 | 86 | 4.45 |  |  |  |
| … family/friends |  |  |  |  |  |  |
| 1 person | 3 | 45 | 6.16 | 0.01 | 2 | 0.995 |
| 2 persons | 5 | 74 | 6.26 |  |  |  |
| 3 or more persons | 6 | 85 | 6.55 |  |  |  |
| psychotherapist or psychologist |  |  |  |  |  |  |
| 1 person | 5 | 43 | 10.3 | 0.63 | 2 | 0.531 |
| 2 persons | 4 | 75 | 5.12 |  |  |  |
| 3 or more persons | 6 | 85 | 6.62 |  |  |  |
| … hotline BAG |  |  |  |  |  |  |
| 1 person | 1 | 47 | 2 | 1.28 | 2 | 0.279 |
| 2 persons | 4 | 75 | 5.04 |  |  |  |
| 3 or more persons | 1 | 89 | 1.13 |  |  |  |
| … had in-patient hospital care |  |  |  |  |  |  |
| 1 person | 5 | 101 | 4.82 | 0.6 | 2 | 0.547 |
| 2 persons | 8 | 170 | 4.43 |  |  |  |
| 3 or more persons | 6 | 228 | 2.73 |  |  |  |
| … in normal care unit or ICU? | ICU | Normal care | % ICU |  |  |  |
| 1 person | 1 | 4 | 19.91 | 0.66 | 2 | 0.517 |
| 2 persons | 1 | 7 | 12.39 |  |  |  |
| 3 or more persons | 0 | 6 | 0 |  |  |  |
| … had a surgical procedure |  |  |  |  |  |  |
| 1 person | 2 | 3 | 40.11 | 0.07 | 2 | 0.929 |
| 2 persons | 3 | 5 | 38.34 |  |  |  |
| 3 or more persons | 3 | 3 | 48.23 |  |  |  |
| … were in rehabilitation clinic |  |  |  |  |  |  |
| 1 person | 1 | 4 | 20.26 | 2.44 | 2 | 0.088 |
| 2 persons | 4 | 4 | 49.92 |  |  |  |
| 3 or more persons | 0 | 6 | 0 |  |  |  |
| … had ambulatory nursing care |  |  |  |  |  |  |
| 1 person | 2 | 3 | 39.63 | 3.15 | 2 | 0.043 |
| 2 persons | 0 | 8 | 0 |  |  |  |
| 3 or more persons | 0 | 6 | 0 |  |  |  |

*The chisquared test of independence was performed with the Rao-Scott second-order correction. The p-values are computed with a Satterthwaite approximation to the distribution and with denominator degrees of freedom as recommended by Thomas and Rao (1990).
